# SEQUENCING OF PLANNED AND UNPLANNED BIRTHS AND IMPLICATIONS FOR MID- AND LATER-LIFE HEALTH AMONG NLSY79 WOMEN

**DOI:** 10.1093/geroni/igac059.1252

**Published:** 2022-12-20

**Authors:** Mieke Thomeer, Clifford Ross, Rin Reczek, Christina Bijou

**Affiliations:** University of Alabama at Birmingham, Birmingham, Alabama, United States; University of Alabama at Birmingham, Birmingham, Alabama, United States; Ohio State University, Columbus, Ohio, United States; Ohio State University, Columbus, Ohio, United States

## Abstract

Existing studies demonstrate that unplanned births (e.g., unwanted, mistimed) are associated with worse health for mothers in the short-term and—according to some preliminary evidence—in mid- and later-life. Yet as life course and reproductive career frameworks highlight, childbearing experiences often unfold over a number of years, with a considerable amount of diversity in pregnancy and birth experiences even for the same individual. For example, a person may have an unplanned birth in late adolescence followed by only planned births in early adulthood. In order to provide a more holistic understanding of how birthing experiences births are associated with midlife health, we use Sequence Analysis (SA) on the 1979 National Longitudinal Survey of Youth (NLSY79; N=3,992) to examine how patterning of planned and unplanned births is associated with physical and mental health at ages 50 and 60 (SF-12). Preliminary analysis indicates that compared to respondents with only planned births, respondents with unplanned birth(s) followed by planned birth(s) have worse physical and mental health at midlife, but there is no difference in health for respondents with only planned births, only unplanned births, and planned birth(s) followed by unplanned birth(s). Future analysis with SA will consider how more detailed sequences (e.g., timing, number and type, ordering, spacing) are associated with these mid- and later-life health outcomes, taking into account selection factors such as childhood SES and educational attainment. This project demonstrates the need for life course perspectives on the long-term health implications of unplanned births, recognizing diversity within and between individuals.

